# Preventing otitis media and respiratory tract infections with the oral probiotic *Streptococcus salivarius* K12

**DOI:** 10.1007/s00431-026-07379-3

**Published:** 2026-09-03

**Authors:** Caroline Halley, Claire Honeywill, Janice Kang, Phillipa Barnes, Gordon Purdie, Gwyneth Steenson, Rae Noble-Adams, Thorsten Stanley, Kathryn Fuge, Lynn McBain, Ben Hudson, Julian Crane

**Affiliations:** 1 Department of Medicine, University of Otago, Wellington, New Zealand; 2 Biostatistical Consulting Group, University of Otago, Wellington, New Zealand; 3 Department of Primary Health Care, University of Otago, Christchurch, New Zealand; 4 Department of Paediatrics and Child Health, University of Otago, Wellington, New Zealand; 5 Bee Healthy Community Oral Health Service, Health New Zealand – Te Whatu Ora, Wellington, New Zealand; 6 Department of Primary Health Care, University of Otago, Wellington, New Zealand

**Keywords:** S*treptococcus salivarius* K12, Probiotic, Acute otitis media, Respiratory tract infections, Infants, Randomised controlled trial

## Abstract

Acute otitis media (AOM) and respiratory tract infections are common in early childhood and are major contributors to antibiotic use. The oral probiotic *Streptococcus salivarius* K12 has demonstrated antimicrobial and immunomodulatory activity in vitro, and preventive effects in children with recurrent AOM. We aim to determine the efficacy of *S. salivarius K12* in preventing AOM in the general infant population. We conducted a multicentre, double-blind, randomised, placebo-controlled trial in Wellington and Christchurch, New Zealand. Children were enrolled at 3–6 months of age, their households were randomised 1:1, and daily study product was given from age 6 to 24 months. The primary outcome was the rate of doctor-recorded AOM. Secondary outcomes included time to first AOM episode, respiratory infections, antibiotic prescribing and healthcare utilisation. Analyses were by intention to treat using mixed-effects negative binomial regression adjusted for region and, for the primary outcome, prior AOM, with random effects for general practice and family. A total of 428 children were randomised by household and 368 (86%) contributed primary outcome data (placebo *n* = 180; active *n* = 188). Doctor-recorded AOM occurred in 70/180 (39%) placebo recipients and 70/188 (37%) probiotic recipients (rate ratio 0.84, 95% CI 0.57–1.24). Time to first AOM episode did not significantly differ (hazard ratio 0.89, 95% CI 0.63–1.26). No significant differences were observed for AOM severity, respiratory infections, antibiotic prescribing or healthcare utilisation. Higher-adherence analyses were also not statistically significant.

*Conclusions:* Daily oral *S. salivarius* K12 did not reduce AOM incidence or respiratory infections in children under 2 years; therefore, routine use for AOM prevention in general paediatric populations is not supported.

*Trial registration:* Australian New Zealand Clinical Trials Registry—ACTRN12618000130268; prospectively registered on 30 January 2018, before enrolment of the first participant on 24 August 2018.

**Table:** 

**What is Known:** • *Streptococcus salivarius K12 is a naturally occurring oral probiotic and has demonstrated antimicrobial and immunomodulatory activity in vitro, and preventive effects in children with recurrent AOM.*	
**What is New:** • *Whether there are preventative effects of daily use for AOM and respiratory infections in infants from the general population.*

**What is Known:**

• *Streptococcus salivarius K12 is a naturally occurring oral probiotic and has demonstrated antimicrobial and immunomodulatory activity in vitro, and preventive effects in children with recurrent AOM.*

**What is New:**

•  *Whether there are preventative effects of daily use for AOM and respiratory infections in infants from the general population.*

## Introduction

Probiotics have been defined by the World Health Organisation as “live microorganisms which when administered in adequate amounts confer a health benefit to the consumer” [1]. Most probiotics derive from natural associations with milk or plants or from the environment [2]. Other probiotics derive from natural human commensals that have been shown to confer a health benefit on natural carriers [3], for example *S. salivarius* which has discreet antimicrobial and immune modulating properties [4]. These microbial properties include inhibitory and bactericidal peptides with activity against pathogens such as *S. pneumoniae*, *H. influenzae* and *M. catarrhalis* [4–6]. Recently in-vitro studies have suggested that some of the anti-bacterial peptides may also have anti-viral properties. For example, they may bind parts of the spike protein of certain viruses thus decreasing cell entry and modulate antiviral immune defences [7].

Respiratory tract infections (RTI), including Acute Otitis Media (AOM), are a major driver of childhood primary care use [8] and antibiotic prescribing in children under 5 years [9, 10]. Given the in-vitro activity of *S. salivarius* it should be an ideal probiotic to reduce Upper Respiratory Tract Infections (URTIs), including AOM which are often viral or bacterial and often specifically involve *S. pneumoniae, H. Influenzae* and *M. catarrhalis.* Three previous clinical trials have reported reductions in AOM incidence and antibiotic prescribing following daily administration of *S. salivarius* K12 in children with recurrent AOM aged 3–10 years [11–13]. However, only one of these involved a double blind randomised controlled trial [11], and none assessed outcomes over more than 3 months. A more recent double-blind, placebo-controlled randomised clinical trial in children aged 2–6 years for a 6-month period did not demonstrate a significant reduction in overall AOM incidence compared with placebo, despite good tolerability and adherence [14]. Collectively, these findings suggest that the effectiveness of *S. salivarius* K12 may be influenced by age, timing of intervention, and underlying disease mechanisms, and that evidence in younger infants—during the period of early immune and microbiome development—remains limited. The objectives of our study were to conduct a double blinded randomised controlled trial to examine the effect of a daily probiotic, *S. salivarius* K12 given to children from age 6 months to 2 years to reduce episodes of AOM and RTI and antibiotic prescribing for RTIs.

## Methods

A two-centre double blind randomised controlled trial enrolled children at 3–6 months of age and administered probiotics or placebo daily from 6 to 24 months in Wellington and Christchurch, New Zealand. The protocol was approved by the Health and Disability Ethics Committee (HDEC 16/CEN/190), overseen by the Health Research Council of New Zealand Data Monitoring Committee (DMC), and prospectively registered with ANZCTR (ACTRN12618000130268).

### Participants and recruitment

Fifteen general practices in the Wellington region and fourteen in the Christchurch region participated in recruitment. Children aged 3–6 months were identified from practice databases, and invitation letters with study information were sent to caregivers via participating practices. Additional participants were recruited through community advertising. Caregivers were screened by telephone, and eligible children were enrolled before the intervention commenced. Randomisation and intervention started at 6 months of age.

Children were eligible if they were aged 3–6 months at enrolment, resided in the wider Wellington or Christchurch regions, and were born at ≥ 28 weeks’ gestation. Exclusion criteria included regular probiotic use (≥ 3 times per week), a history of severe milk or soy allergy, declined consent for access to medical records, significant cardiac disease, immunosuppressive illness or medication use, or concurrent participation in other health research studies. Children commenced the assigned study product at 6 months of age and were followed for the 18-month intervention period until age 24 months; no post-intervention follow-up was conducted. Recruitment concluded in May 2020, and follow-up and data collection continued until late 2021.

### Randomisation and blinding

Randomisation was conducted at the family/household level using a computer-generated permuted block sequence with randomly varying block sizes up to 10, stratified by region. Pre-prepared study packs containing either active or placebo product were labelled with a unique study identification number and allocated sequentially in order of enrolment. Eligible children from the same household were allocated to the same treatment group. Participants, caregivers, investigators, and outcome assessors were blinded to treatment allocation throughout the study.

### Intervention

Active and placebo powders were encapsulated and contained identical excipients (trehalose, lactitol, maltodextrin, a small quantity of isomalt, and natural strawberry flavouring), with appearance, smell, taste and packaging identical. The active formulation additionally contained freeze-dried *Streptococcus salivarius* K12, providing at least 1.25 × 10⁹ colony-forming units (CFU) per dose at the date of manufacture. Study products were manufactured, bottled, and labelled by BLIS Technologies Ltd. 

Participants began one dose daily at 6 months of age and continued for 18 months, until age 24 months. Study product was supplied in three 6-month allocations: at enrolment and at 6 and 12 months after intervention commencement. Each dose was mixed with a small amount of water to make a paste applied to the mouth and gums, direct application to food or application of dry powder to the teeth and gums following oral cleaning, as per the manufacturer’s instructions.

### Storage, handling, and product integrity

Study products were stored at ≤ 4 °C prior to distribution. Bottles exposed to heat were replaced. During use, bottles were stored at room temperature for up to 2 months provided they were not exposed to heat. Replacement bottles were provided where product was lost, overheated, or substantially spilled. Probiotic viability was periodically tested by the manufacturer on returned bottles.

### Protocol and adherence monitoring

Participants received text message reminders to start and stop the administration of the powder, and follow-up telephone questionnaires to assess adherence. Returned bottles were collected by courier or at study visits and weighed. Differences between pre- and post-use weights were used as an objective measure of adherence.

### Outcomes

The primary outcome was the rate of doctor-recorded AOM during the 18-month intervention period. Secondary outcomes included the number of medical visits for respiratory tract infections (RTIs), doctor prescriptions of antibiotics for RTI or OM, number of doctor diagnoses of croup, and severity of doctor-diagnosed OM in terms of bilateral disease, visible discharge from one or both ears, bilateral discharge, and referral to ear, nose and throat (ENT) services. Other secondary outcomes were the frequency of doctor-diagnosed RTI and dental caries identified from Community Oral Health records and assessed by a dental public health specialist for Wellington participants. Parent-reported outcomes included ear infections, RTIs, hospital admissions for respiratory conditions, outpatient referrals to paediatrics or ENT services, and antibiotic courses for respiratory illness.

### Outcome ascertainment and GP record review

Outcome data were obtained from caregiver questionnaires and structured review of general practice records. General practice records were reviewed for the prespecified 18-month period that the participants were taking the powder only, and events occurring after the individual participant’s scheduled completion date were not included. Unscheduled GP visits for illness were classified as consultations and reviewed using a dedicated electronic data capture system. Study clinicians identified AOM, RTI, croup and associated antibiotic prescribing. Analyses used all participants with data available for the relevant outcome: GP-record outcomes required retrievable GP records, whereas parent-reported outcomes used completed caregiver questionnaires. Consequently, denominators differed between outcome types. No outcome data were imputed.

### Adverse events

Adverse events were captured through scheduled questionnaires and additional reporting as required. Serious adverse events were reviewed by the DMC.

### COVID-19–related protocol modifications

Protocol modifications were implemented in response to COVID-19 public health restrictions. COVID-19 restrictions required remote enrolment (*n* = 30) or follow-up for some participants, couriered study product, delayed bottle return, and use of parental report or clinical records for anthropometric data. Oral mucosal swabs could not be collected at the end-of-study visits.

### Sample size

Based on New Zealand estimates of 499 AOM episodes per 1000 child-years in children aged under 1 year and 377 per 1000 child-years in children aged 1–2 years [15], the expected rate from age 6 to 24 months was 586 episodes per 1000 children. A total sample of 420 participants provided 90% power at the 5% significance level to detect a 50% reduction in this rate using an unclustered negative binomial regression, assuming the reported distribution of multiple episodes [15]. A 50% reduction was selected as a clinically meaningful effect and was smaller than the 70% reduction reported in an earlier non-randomised, non-placebo-controlled and unblinded study [12]. No allowance was made for attrition because withdrawal of consent for the GP-record search was initially expected to be rare.

### Statistical analysis

Analyses were intention-to-treat. Event rates were compared using mixed-effects negative binomial regression adjusted for region, with random effects for family and general practice; primary AOM was additionally adjusted for prior ear infection. Analyses used SAS 9.4 (GLIMMIX). Fisher’s exact test was used when one group had no events and all events occurred in different families. Time to first AOM was analysed using a Cox proportional hazards model. Available outcome data were analysed without imputation; secondary outcomes were exploratory with no adjustment for multiple comparisons. Higher-adherence analyses used the same model specifications except where indicated. A post hoc sensitivity analysis compared prior GP- or nurse-practitioner-diagnosed ear infection by GP-record availability using Fisher’s exact test and logistic regression adjusted for treatment group and region; interactions assessed differences by treatment group and region.

## Results

**Fig. 1:**
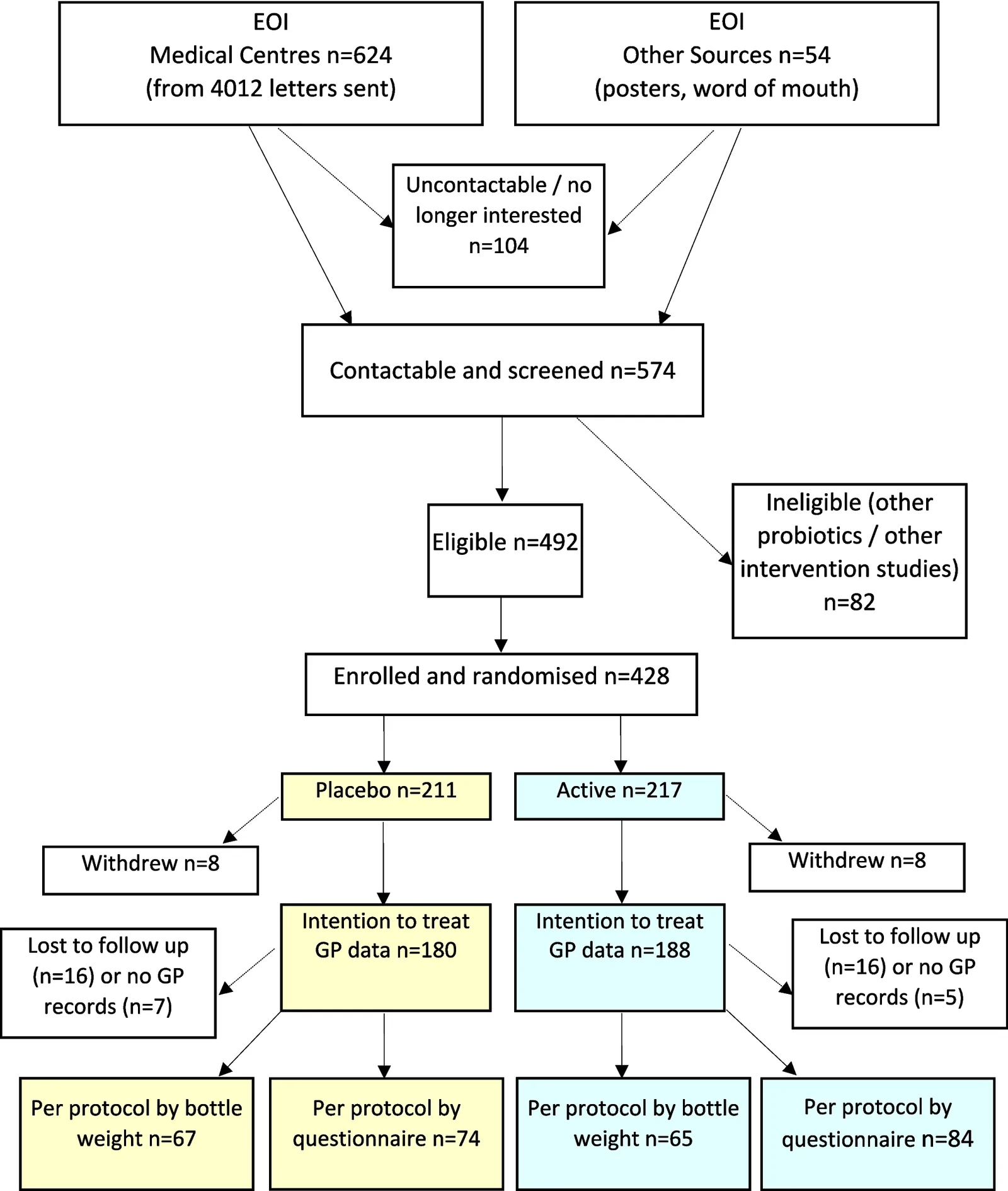
Participant flow diagram. EOI, expression of interest. GP-data numbers show the primary-outcome population; denominators for parent-reported outcomes vary according to questionnaire availability (Table 2)

**Fig. 2:**
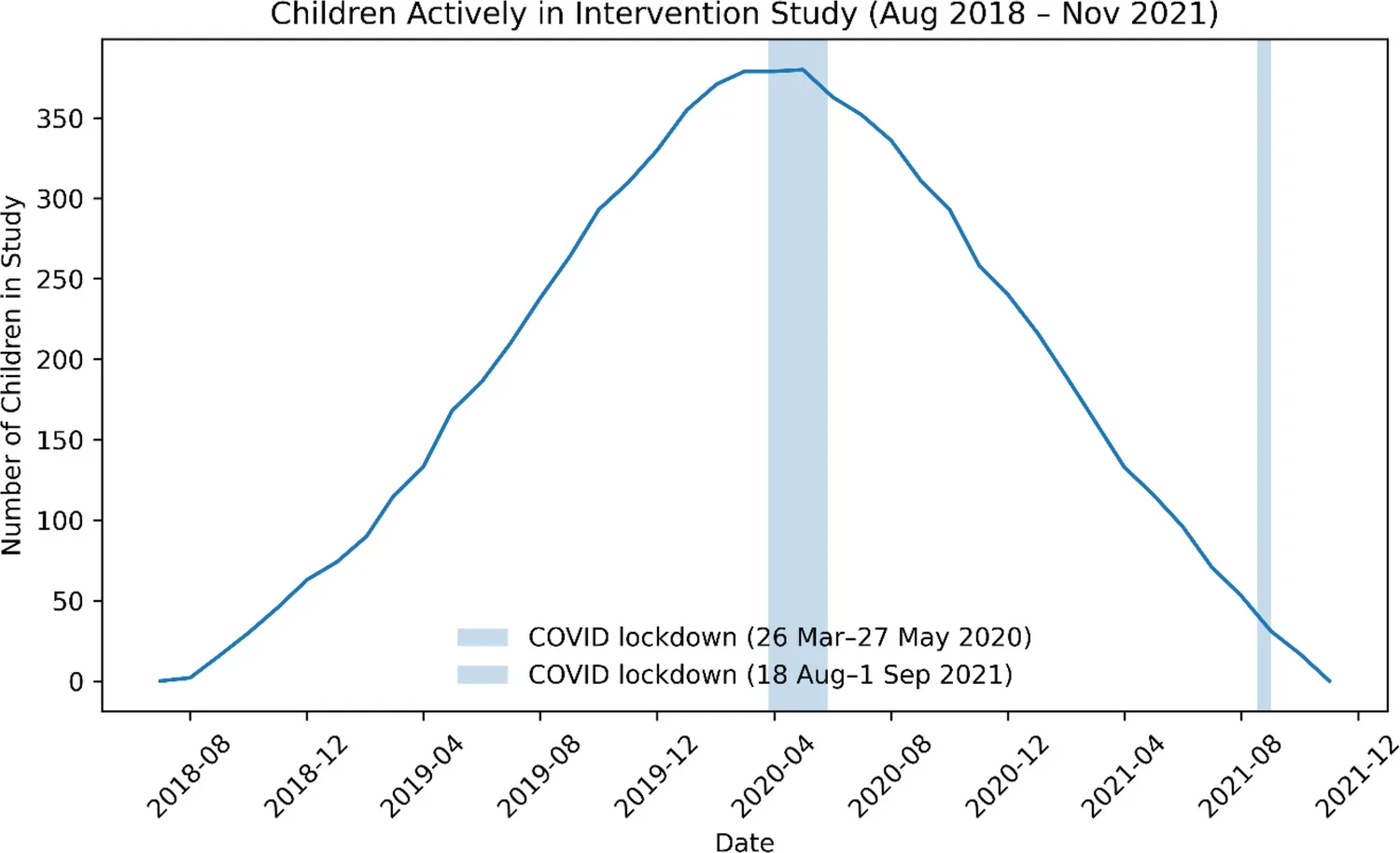
Timeline of participation in the study—highlighting COVID-19 restrictions

Progress through the study is shown in Figs. 1 and 2. Four hundred twenty-eight children were enrolled, 210 in Wellington and 218 in Christchurch, 368 (86%) had retrievable GP outcome data after the planned 18-month intervention and follow-up period. Recruitment ended in May 2020, while intervention follow-up continued until late 2021. Baseline characteristics were broadly similar between groups (Table 1), although the active group had numerically more prior ear infection (8.8% vs 5.2%) and prior antibiotic treatment for ear infection (6.0% vs 2.4%).

**Table 1:** Baseline characteristics

Characteristic	Placebo (*n* = 211)	Active (*n* = 217)
Female, *n* (%)	101 (47.9%)	105 (48.4%)
Gestational age, weeks, median (IQR)	39.7 (38.6–40.6)	39.6 (38.6–40.4)
Caesarean delivery, *n* (%)	57 (27.0%)	73 (33.8%)
Birth weight, kg, median (IQR)	3.5 (3.2–3.8)	3.5 (3.2–3.9)
Prior ear infection	11 (5.2%)	19 (8.8%)
Prior bronchiolitis	15 (7.1%)	22 (10.1%)
Prior other chest infection	7 (3.3%)	9 (4.1%)
Prior croup	4 (1.9%)	9 (4.1%)
Prior antibiotics for ear infection	5 (2.4%)	13 (6.0%)
Total courses of antibiotics for ear infection, *n*	8	16
Prior ENT referral	7 (3.3%)	4 (1.8%)
Eczema	67 (31.8%)	62 (28.6%)
Maternal smoking in pregnancy	9 (4.3%)	7 (3.2%)
Household smoking	1 (0.5%)	1 (0.5%)
Breastfed to 6 months of age	164 (77.7%)	167 (77.0%)
Sibship (half and full siblings)	131 (62.1%)	138 (63.6%)
Pneumococcal vaccination	198 (93.8%)	196 (90.3%)

Values are *n* (%) unless otherwise stated; gestational age and birth weight are median (interquartile range). Gestational age, delivery mode and birth weight were available for 216 participants in the active group.

**Fig. 3:**
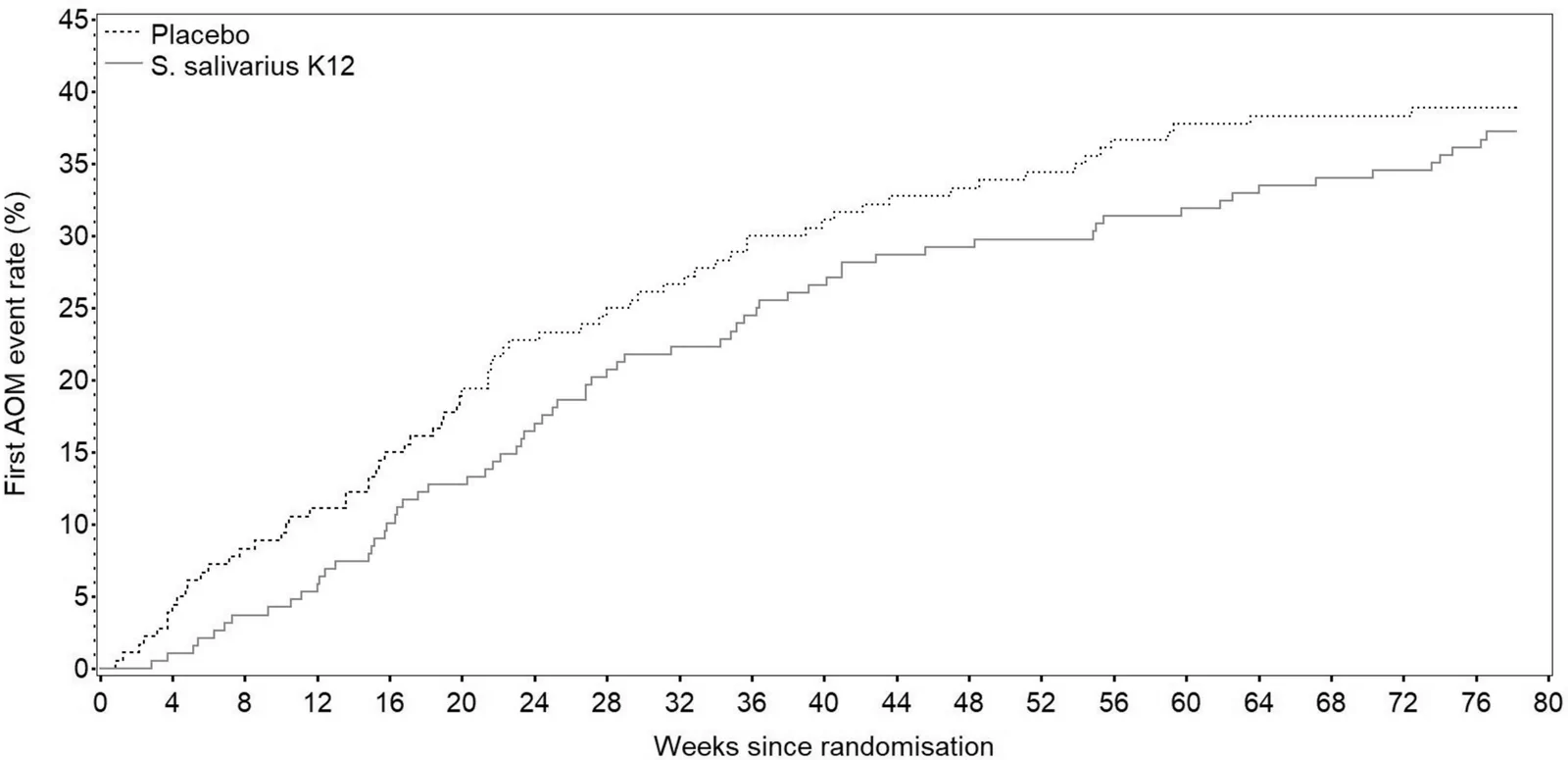
Percentage first doctor diagnosed episode of acute otitis media. (Hazard ratio 0.89 (95% CI 0.63,1.26) *p* = 0.51)

Table 2 shows the primary and secondary outcomes. Doctor-recorded AOM occurred in 70/180 placebo participants (129 episodes) and 70/188 active participants (109 episodes), with no significant difference in event rates (adjusted rate ratio 0.84, 95% CI 0.57–1.24; *p* = 0.32). In a post hoc sensitivity analysis, prior GP- or nurse-practitioner-diagnosed ear infection was more common among children without available GP notes (10.9% vs 5.1%), but the adjusted association was not statistically significant (OR 2.40, 95% CI 0.91–6.35; *p* = 0.078), with no evidence of interaction by treatment group or region. No secondary outcome differed significantly between groups. Time to first doctor-diagnosed AOM also did not differ significantly (hazard ratio 0.89, 95% CI 0.63–1.26; *p* = 0.51; Fig. 3).

**Table 2:** Primary and secondary outcomes

	Placebo Percent (n/N) Total episodes	*S. salivarius* K12 Percent (n/N) Total episodes	K12/placebo rate ratio (95% CI) p*
Primary outcome			
Number of episodes of doctor reported AOM	70/180 (39%); 129	70/188 (37%); 109	0.84 (0.57, 1.24) *p* = 0.32
Secondary outcomes			
Severity of doctor diagnosed OM—Bilateral OM	46/180 (26%); 80	49/188 (26%); 83	1.02 (0.62, 1.68) *p* = 0.93
- Discharge OM	10/180 (6%); 18	6/188 (3%); 7	0.60 (0.17, 2.10) *p* = 0.35
- Bilateral discharge OM	3/180 (2%); 4	0/188 (0%); 0	*p* = 0.12†
- ENT referral	7/180 (4%); 9	2/188 (1%); 2	0.29 (0.04, 2.19) *p* = 0.19
Number of medical visits for Upper and Lower Respiratory Tract Infections (composite)	48/180 (27%); 79	55/188 (29%); 89	1.08 (0.61, 1.91) *p* = 0.76‡
Doctor prescriptions for antibiotics for respiratory conditions (OM, URTI, LRTI)	74/180 (41%); 187	83/188 (44%); 165	0.91 (0.61, 1.36) *p* = 0.57
Number of doctor diagnoses with croup	31/180 (17%); 39	27/188 (14%); 45	1.10 (0.49, 2.47) *p* = 0.77‡
Number of GP visits for OM	75/180 (42%); 173	75/188 (40%); 163	0.93 (0.62, 1.40) *p* = 0.68
Frequency of doctor diagnosed RTI	48/180 (27%); 72	55/188 (29%); 81	1.08 (0.64, 1.82) *p* = 0.74
Number with dental caries at 2 years of age	0/77 (0%)	0/74 (0%)	not estimable as zero events
Parental reported ear infections	71/192 (37%); 167	72/196 (37%); 151	0.93 (0.62, 1.38) *p* = 0.68
Parental reported RTI	188/192 (98%); 1552	193/196 (98%); 1763	1.05 (0.90, 1.22) *p* = 0.46
Parental reports of hospital admissions for respiratory conditions (OM, URTI, LRTI)	7/192 (4%); 7	8/196 (4%); 14	1.19 (0.33, 4.30) *p* = 0.76
Parental reports of outpatient referrals to paediatrics or ENT for respiratory tract infections (OM, URTI, LRTI)	12/170 (7%); 31	7/179 (4%); 27	0.83 (0.11, 6.04) *p* = 0.83
Parental report of course of antibiotics taken for respiratory conditions (OM, URTI, LRTI)	80/192 (42%); 205	83/196 (42%); 175	0.90 (0.62, 1.30) *p* = 0.52

Values are n/N (%) and total episodes, where applicable.

*Mixed-effects negative binomial regression adjusted for region, with random effects for family and medical centre; primary AOM also adjusted for prior ear infection.

†Fisher’s exact test.

‡Region and medical-centre terms omitted.

The powder was well tolerated. Parent-reported serious adverse events (hospitalisation for ≥ 24 h) did not differ between groups (19/211 placebo; 27/217 active; *p* = 0.60) and were not considered related to study product by the DMC.

Self-reported adherence did not differ between groups. The proportion taking powder on at least 5 days per week fell from approximately two-thirds in the first 6 months to around half at 12–18 months (all *p* ≥ 0.75). Bottle returns were also similar (*p* = 0.67); 28.4% of placebo and 30.0% of active participants returned no bottles, while 27.0% and 26.3%, respectively, returned all nine. Powder use was nearly identical (median 204.9 g placebo vs 202.2 g active; *p* = 0.95).

Among those participants with higher adherence (Table 3), point estimates for the primary AOM outcomes favoured *S. salivarius* K12, including lower proportions and rates of doctor-recorded AOM and fewer GP visits for OM than in the placebo group. However, these differences were not statistically significant and the confidence intervals were wide.

**Table 3:** Primary outcome amongst those with greater treatment compliance

	Placebo Percent reported, total,	*S. salivarius* K12 Percent reported, total,	K12/placebo rate ratio (95% CI) p*
Primary outcome			
Number of episodes of doctor reported AOM	28/74 (38%); 58	25/84 (30%); 38	0.59 (0.28, 1.27) *p* = 0.13
Secondary outcomes – severity of AOM			
Severity of doctor diagnosed OM - Bilateral OM	16/74 (22%); 35	16/84 (19%); 27	0.86 (0.31, 2.35) *p* = 0.70
- Discharge OM	5/74 (7%); 7	2/84 (2%); 3	0.65 (0.07, 5.90) *p* = 0.62
- Bilateral discharge OM	1/74 (1%); 1	0/84 (0%); 0	*p* = 0.47†
- ENT referral	3/74 (4%); 3	1/84 (1%); 1	0.45 (0.02, 11.85) *p* = 0.53‡
Number of GP visits for OM	29/74 (39%); 79	26/84 (31%); 61	0.74 (0.35, 1.61) *p* = 0.34

Values are participants with at least one event/participants in the higher-adherence subgroup (%) followed by total episodes.

*Mixed-effects negative binomial regression.

†Fisher’s exact test with one observation per family.

‡No adjustment for centre.

## Discussion

This randomised, double-blind, placebo-controlled trial found no evidence that daily *S. salivarius* K12 from age 6 months to 2 years reduced AOM, other RTIs or antibiotic prescribing. Findings were consistently null across intention-to-treat outcomes, including time to first AOM, severity and healthcare utilisation. Higher-adherence estimates favoured *S. salivarius* K12 but were imprecise and not statistically significant.

Our results are consistent with the placebo-controlled trial by Sarlin et al., which also found no significant reduction in AOM [14]. Together, these trials suggest that oral *S. salivarius* K12 does not substantially reduce AOM risk in otherwise healthy children.

Earlier clinical studies reporting reductions in AOM incidence largely involved children with recurrent disease and often used alternative delivery routes, including intranasal administration of streptococcal strains [11–13]. These interventions may act through local competitive colonisation of the nasopharynx rather than systemic or oral immune modulation. In addition, several earlier studies were small, short in duration, or lacked placebo control, limiting confidence in the magnitude or generalisability of reported effects. Systematic reviews of probiotics for ear and respiratory outcomes in children have reported modest overall reductions in AOM incidence, but interpretation is complicated by substantial heterogeneity in probiotic strains, delivery methods, treatment duration, and study quality [16, 17]. Many studies include mixed probiotic formulation. Reviews of *S. salivarius* K12 specifically have included small, short-duration or non-randomised studies with limited control groups [18, 19]. More recent meta-analysis of probiotic, prebiotic and symbiotic interventions suggests modest reductions in AOM incidence overall but highlights heterogeneity and variable risk of bias across studies [20]. Our findings from a large, well-controlled trial therefore provide important strain-specific evidence regarding oral *S. salivarius* K12 in a general infant population.

The absence of measurable clinical benefit is notable given the well-described antimicrobial and immunomodulatory properties of *S. salivarius* K12 demonstrated in vitro [7]. Although mucosal colonisation following oral administration of K12 has been documented in several studies, colonisation appears to be variable between individuals and often transient rather than stable [14, 21]. Detectable carriage may occur shortly after administration, but persistence over longer periods is inconsistent, and sustained ecological modification of the nasopharyngeal microbiota has not been reliably demonstrated. As a result, the presence of the organism on mucosal surfaces may not be sufficient in duration or magnitude to meaningfully alter pathogen transmission dynamics or host susceptibility to infection.

The infant nasopharyngeal microbiome undergoes rapid ecological change, with colonisation influenced by host immunity, viral exposure, antibiotic use and environmental factors [22, 23]. AOM pathogenesis is multifactorial, involving viral-bacterial interactions, Eustachian tube function, host immune maturation and microbial community dynamics [24]. Targeting a limited number of bacterial pathogens and transient augmentation may be an insufficient way to alter disease risk if viral triggers or host susceptibility dominate.

This study has several important strengths. It was a large, multicentre, double-blind, placebo-controlled randomised trial with prolonged follow-up, and 86% of randomised children contributed GP-record data for the primary outcome. Outcome ascertainment incorporated structured review of general practice medical records, reducing reliance on parental recall. Objective measures of adherence were obtained through bottle-weight monitoring and specific questionnaire assessment, and product viability was periodically confirmed. The intervention covered a substantial period of early childhood, when AOM risk is high and preventive strategies are most clinically relevant. Together, these design features strengthen the evidence regarding the lack of efficacy of oral *S. salivarius* K12 in this age group.

Several limitations should be considered. COVID-19 public health restrictions substantially altered respiratory pathogen circulation and healthcare utilisation worldwide and in New Zealand specifically. Population lockdowns implemented to control SARS-CoV-2 transmission resulted in marked reductions in influenza and other respiratory viruses, including near-elimination during periods of strict population quarantine measures [25]. Paediatric healthcare utilisation and diagnoses of infectious diseases also declined substantially during lockdown periods, including documented reductions in acute otitis media presentations [26–28]. New Zealand studies demonstrated dramatic reductions in infant respiratory hospitalisations during the elimination strategy period [29].

The first national lockdown occurred when trial participation was at its peak (around 380 active participants) and therefore had the greatest potential to reduce exposure to respiratory pathogens or alter healthcare-seeking behaviour. In contrast, the second lockdown occurred late in follow-up when relatively few children remained active (approximately 50) and is less likely to have materially influenced overall results.

The observed AOM rate was broadly consistent with the magnitude anticipated in the sample-size calculation, despite pandemic disruptions. However, COVID-19-related changes may have nevertheless reduced opportunities to observe differences between treatment groups.

Other limitations may have reduced precision or introduced bias. GP outcome data were available for 368 rather than the planned 420 children; 60 (14%) lacked primary outcome data, mainly because of record-retrieval and reconsent difficulties during COVID. Complete-case analysis could be biased if record availability related to infection risk or treatment response. Prior GP- or nurse-practitioner-diagnosed ear infection was more common among children without GP notes, suggesting missingness may not have been independent of baseline AOM risk, although the estimate was imprecise and did not differ by treatment allocation or region. The study was powered to detect a 50% reduction and may have missed a more modest effect. Baseline prior ear infection and antibiotic-treated ear infection were numerically higher in the active group, but adjustment for prior ear infection did not materially alter the treatment estimate.

Adherence declined over time, with approximately two-thirds taking powder on at least 5 days per week initially and around half by 12–18 months. This reflects the difficulty of sustaining a daily preventive treatment for 18 months. Higher-adherence estimates favoured *S. salivarius* K12 but were imprecise and should be interpreted cautiously.

### Clinical implications

Taken with existing placebo-controlled evidence, our findings do not support routine oral *S. salivarius* K12 for prevention of AOM or RTIs in the general population of infants and young children. The decline in adherence later in the study also suggests that long-term daily powder administration may be difficult for families over prolonged periods.

### Future research directions

Further research would clarify whether probiotic interventions have a role in selected populations or under different conditions. Priorities should include children at high baseline risk of recurrent AOM; alternative delivery routes that enhance nasopharyngeal colonisation; mechanistic studies of colonisation persistence and microbial community effects; and earlier or longer interventions during microbiome development. Studies should also examine reasons for lower adherence and strategies to improve it. Understanding host–microbe interactions in early childhood and infancy and identifying populations most likely to respond may help determine whether targeted microbial prevention can meaningfully reduce respiratory disease burden.

## Conclusions

In this large randomised trial, oral *S. salivarius* K12 did not significantly reduce AOM, RTIs or antibiotic prescribing in children aged 6 months to 2 years. These findings do not support routine use for AOM prevention in general paediatric populations.

## Data Availability

The data generated by this study will not be shared with the public or other investigators as permission was not sought from the participants.
